# Independent and Joint Associations of Socioeconomic Status and Dietary Quality with Depressive and Anxiety Symptoms Among Chinese Older Adults: A Cross-Sectional Study of the Chinese Longitudinal Healthy Longevity Survey

**DOI:** 10.3390/nu18213540

**Published:** 2026-10-27

**Authors:** Jianghua Huo, Chongyu Ding, Xueyan Zhang, Xiangwei Li

**Affiliations:** 1 School of Public Health and Management, Jiangsu Medical College, Yancheng 224005, China; 2 School of Global Health, Chinese Center for Tropical Diseases Research, Shanghai Jiao Tong University School of Medicine, Shanghai 200025, China; 3 Hainan International Medical Center, Shanghai Jiao Tong University School of Medicine, Qionghai 571434, China

**Keywords:** socioeconomic status, dietary quality, depressive symptoms, anxiety symptoms, Chinese Longitudinal Healthy Longevity Survey, anti-inflammatory dietary diversity

## Abstract

Objectives: We assessed how socioeconomic status (SES) and diet quality, separately and jointly, related to depressive and anxiety symptoms in older Chinese adults. Methods: Data came from 9278 participants aged 65 years or older in the 2018 Chinese Longitudinal Healthy Longevity Survey. Education, main occupation before age 60, and annual household income were integrated into latent SES classes. Diet quality was represented by the Chinese-modified Mediterranean-DASH Intervention for Neurodegenerative Delay score (cMIND), Healthy Eating Index (HEI), Dietary Diversity Score (DDS), and Anti-Inflammatory Dietary Diversity Score (AIDDS). Depression and anxiety symptom status was determined from the 10-item Center for Epidemiologic Studies Depression Scale (CES-D-10) and 7-item Generalized Anxiety Disorder Scale (GAD-7). We used multivariable logistic models, joint-exposure models, restricted cubic splines, exploratory SES-by-diet interaction models, and multiple imputation for missing covariates. Results: Relative to high SES, low SES was associated with greater odds of depressive symptoms (OR = 1.50, 95% CI: 1.32–1.71) and anxiety symptoms (OR = 1.76, 95% CI: 1.43–2.16). All four dietary indices showed inverse associations with both outcomes. The largest joint estimates occurred for low SES plus low AIDDS (depressive symptoms: OR = 2.26, 95% CI: 1.94–2.64; anxiety symptoms: OR = 2.69, 95% CI: 2.12–3.40). No overall multiplicative interaction was found for dichotomized diet scores. For standardized continuous scores, nominal SES-by-diet interactions were observed for HEI, DDS, and AIDDS for depressive symptoms, but after Benjamini-Hochberg false-discovery-rate (FDR) correction only DDS and AIDDS remained below q < 0.05; no anxiety interaction survived correction. Conclusions: Socioeconomic disadvantage and poorer diet quality were each associated with greater symptom burden, while their co-occurrence identified the least favorable joint profile. Longitudinal and intervention studies are needed before dietary improvement can be interpreted as reducing SES-related mental-health inequalities.

## 1. Introduction

Depressive and anxiety symptoms contribute substantially to poor health in later life through their associations with impaired functioning, lower quality of life, and premature mortality [1,2,3,4,5,6]. Their growing population burden has made mental health in older age an increasingly important public-health concern. Vulnerability may be amplified when age-related functional loss, multimorbidity, social isolation, bereavement, and financial insecurity occur together [4,5,6]. Identifying modifiable factors that accompany socioeconomic disadvantage may therefore help characterize groups at elevated risk and inform healthy-aging strategies.

Socioeconomic status (SES) reflects access to educational, occupational, and material resources and is a major social determinant of health [7,8,9,10,11,12,13,14]. Across aging cohorts, lower SES has been linked prospectively to more depressive symptoms, while cross-sectional evidence also indicates socioeconomic gradients in anxiety [10,11,12,14,15]. Diet is another potentially modifiable correlate of mental health. Both longitudinal and cross-sectional studies have related healthier dietary patterns to less depression and anxiety [16,17,18,19,20,21,22]. Among older Chinese adults, work using the Chinese Longitudinal Healthy Longevity Survey (CLHLS) has reported favorable mental-health associations for the Chinese-modified Mediterranean-DASH Intervention for Neurodegenerative Delay score (cMIND), Healthy Eating Index (HEI), Dietary Diversity Score (DDS), and Anti-Inflammatory Dietary Diversity Score (AIDDS) [18,19,20,23].

These social and behavioral domains are unlikely to operate in isolation. Limited economic resources can restrict the affordability, availability, and range of foods that older adults are able to choose [13,24,25]. CLHLS analyses also suggest that health behaviors cluster; for example, participants combining sufficient dietary diversity with current exercise had the most favorable depressive and anxiety profiles among the joint behavior categories evaluated [26]. Even so, most prior work has considered SES and diet separately or has relied on a single dietary construct. It remains unclear whether several related dietary indices show a consistent joint pattern with SES and whether any descriptive joint pattern is accompanied by statistical interaction. Examining both questions is important because co-occurrence of two adverse exposures is not equivalent to multiplicative synergy.

We therefore used the 2018 CLHLS to address three questions: whether SES and each of four dietary indices were independently associated with depressive and anxiety symptoms; whether combinations of SES and diet quality identified groups with different symptom burdens; and whether SES modified the diet-symptom associations on the multiplicative scale. We expected lower SES and poorer diet quality to be associated with less favorable outcomes and anticipated the greatest symptom burden among participants exposed to both disadvantages.

## 2. Materials and Methods

### 2.1. Study Population

**Figure 1:**
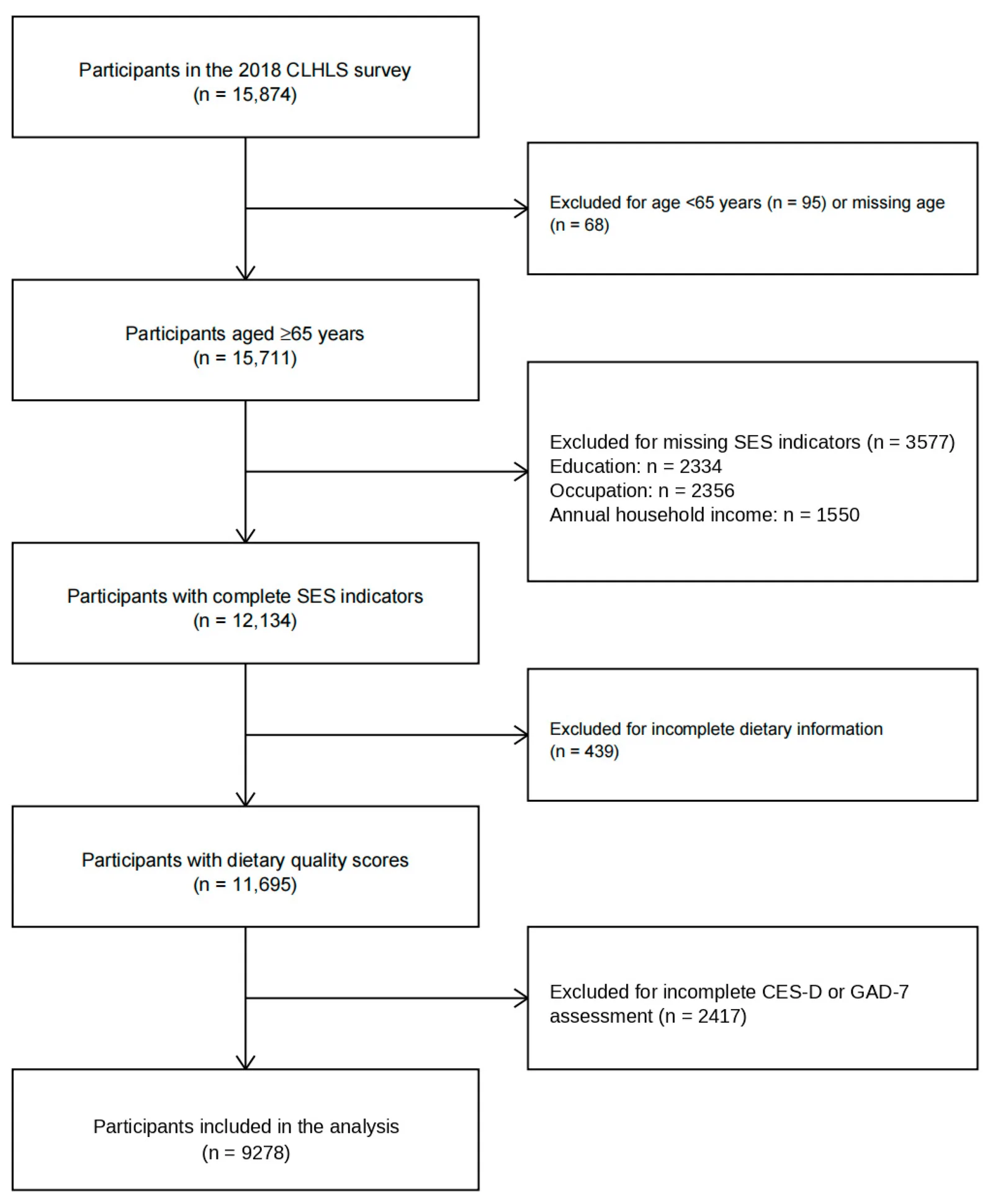
Selection of the analytic sample from the 2018 Chinese Longitudinal Healthy Longevity Survey (CLHLS). CES-D, Center for Epidemiologic Studies Depression Scale; GAD-7, 7-item Generalized Anxiety Disorder Scale; SES, socioeconomic status.

The analytic cohort was assembled from the 2018 CLHLS, a nationwide aging survey covering 23 Chinese provinces and using multistage sampling with interviewer-administered assessments [27,28]. The starting sample contained 15,874 respondents. We sequentially removed 95 people younger than 65 years, 68 with no age information, 3577 lacking at least one SES indicator, 439 without sufficient dietary information to calculate the diet scores, and 2417 without complete depressive- or anxiety-symptom assessments. After these exclusions, 9278 adults aged at least 65 years remained for analysis (Figure 1). Supplementary Table S1 contrasts the characteristics of included and excluded respondents. The cited ethics approval (IRB00001052-13074) covered the broader CLHLS protocol and was granted by the Biomedical Ethics Committee of Peking University; written informed consent was obtained from every participant. The present study was a secondary analysis of the 2018 CLHLS wave and involved no additional participant recruitment or data collection.

### 2.2. Assessment of Socioeconomic Status

SES classes were estimated from three individual-level indicators: educational attainment, annual household income, and the participant’s main occupation before age 60 [7,8,9]. Education was coded as 0 years, 1–5 years, 6–12 years, or at least 13 years. Household income was grouped into ≤10,000, >10,000 to ≤50,000, and >50,000 yuan per year. Pre-60 occupation was classified as formal work (professional/technical, government or institutional/managerial, commercial/service/industrial, military, or self-employed work) or informal work (farming, homemaking [unpaid domestic work], and other work) [13]. We compared one- through five-class latent class analysis (LCA) solutions using AIC, BIC, the likelihood-ratio G^2^ statistic, class size, and classification diagnostics (Supplementary Table S2). The three-class model minimized AIC and BIC while retaining interpretable profiles. Classes were designated low, middle, and high SES according to the distributions of the three indicators (Supplementary Table S3).

### 2.3. Assessment of Dietary Quality

Dietary intake frequency was obtained from the abbreviated CLHLS food-frequency questionnaire, which reflects commonly consumed foods in China and has been used in nutritional epidemiology [29]. We operationalized diet quality with four related but differently weighted measures: cMIND, HEI, DDS, and AIDDS. For every index, a larger score represented a more favorable dietary profile.

The cMIND score incorporated 12 components: staple-food type and amount, edible oil, fresh vegetables and fruit, mushrooms or algae, fish, legumes and legume products, nuts, garlic, tea, and sugar [18,19,30,31]. Most components contributed 0, 0.5, or 1 point; the staple-food and oil components contributed either 0 or 1 point. Summing the components yielded a score from 0 to 12, with larger values denoting closer adherence to the cMIND pattern.

HEI comprised 13 food groups: vegetables and fruit; meat, fish, and eggs; legumes and legume products; dairy products; tea, garlic, nuts, and mushrooms or algae; and the less favorable components sugar and salt-preserved vegetables [20,23]. Fruit and vegetable frequency was scored from 0 to 3, whereas the other groups used 0–4 points across five intake-frequency categories. The sugar and salt-preserved vegetable components were reverse coded. The resulting HEI ranged from 0 to 50, with higher totals indicating a healthier pattern.

DDS covered nine categories: fresh vegetables, fresh fruit, meat, fish, eggs, legumes and legume products, dairy products, nuts, and tea [32,33]. Each category meeting its prespecified regular-consumption criterion contributed one point, producing a 0–9 total. AIDDS focused on five anti-inflammatory groups (vegetables, fruit, legumes and legume products, nuts, and tea) and ranged from 0 to 5 [18,32].

### 2.4. Assessment of Depressive and Anxiety Symptoms

Depressive symptoms were measured with the 10-item Center for Epidemiologic Studies Depression Scale (CES-D-10), which has previously been used in the CLHLS [16,19]. Each response contributed 0–3 points according to symptom frequency, with the positively phrased items scored in the opposite direction. The summed scale ranged from 0 to 30. We classified participants scoring at least 10 as having depressive symptoms [16,34].

Anxiety symptoms were measured with the seven-item Generalized Anxiety Disorder scale (GAD-7). Item responses ranged from 0 (never) to 3 (almost every day), giving a possible total of 0–21. A score of 5 or above was used to identify the presence of anxiety symptoms [16,18].

### 2.5. Covariates

Adjustment variables were prespecified from prior literature and their availability in the CLHLS. Age, sex, urban/rural residence, and marital status represented sociodemographic characteristics. Smoking, alcohol use, and exercise were each recorded as never, former, or current from self-report at interview. Body mass index (BMI) was calculated from measured height and weight and grouped as <18.5, 18.5–<24.0, or ≥24.0 kg/m^2^. Hypertension, diabetes, heart disease, stroke or other cerebrovascular disease, and respiratory disease were coded as present when the participant reported a physician diagnosis or use of medication for that condition. We included these prevalent conditions because they can affect both dietary behavior, including clinician-advised restrictions, and mental-health symptoms; their temporal position cannot be resolved in a cross-sectional analysis. Cognitive function was represented by the Chinese version of the Mini-Mental State Examination (CMMSE; 0–30, higher scores indicating better performance) [16]. Functional status was summarized with a six-item activities of daily living (ADL) disability score covering eating, dressing, transferring, toileting, bathing, and continence [35,36]. Social isolation was represented by a composite based on social engagement, living arrangement, social network, and social support; higher values indicated greater isolation [37].

### 2.6. Statistical Analysis

For descriptive analyses, categorical characteristics are reported as counts and percentages and continuous measures as means with standard deviations (SD) within each SES class. Differences across SES classes were tested with chi-square tests or one-way analysis of variance (ANOVA), as appropriate. Correlations among cMIND, HEI, DDS, and AIDDS were displayed as a pairwise correlation matrix (Supplementary Figure S1).

Binary logistic regression provided odds ratios (ORs) with 95% confidence intervals (CIs) for each mental-health outcome. Model 1 contained no covariates; Model 2 included age, sex, residence, and marital status; and Model 3 further included smoking, drinking, exercise, BMI, the five chronic disease indicators, CMMSE, ADL disability, and social isolation. Each diet score was examined per one-point increase, above versus below its imputation-specific median, and by quartile. We additionally fitted restricted cubic splines with the Model 3 covariates to examine dose-response shape, reporting tests for the overall association and for departure from linearity (Supplementary Figure S2).

For joint-exposure analyses, we crossed the three SES classes with the high/low category of each dietary index, creating six combinations per index. The high-SES/high-diet group was the comparator. Model 3 covariates were included in the corresponding logistic regressions. These six-category estimates were used to describe co-occurring exposures and were not interpreted as interaction tests.

We assessed multiplicative effect modification by entering SES-by-diet product terms into Model 3. Separate models used either median-dichotomized diet scores or standardized continuous scores expressed per 1-SD increase; an overall interaction *p* value summarized evidence across SES categories (Supplementary Tables S9 and S10). Robustness analyses comprised complete-case models and analyses stratified by age (<80 or ≥80 years) and sex. Stratified models included the Model 3 adjustment set except for the variable defining the stratum (Supplementary Tables S5–S8).

Multiple imputation by chained equations was used only for missing covariates after the analytic cohort had been fixed on complete age, SES indicators, dietary measures, and outcome assessments. We did not impute missing SES indicators, dietary measures, or outcome scales because these variables defined analytic eligibility and the derived exposure/outcome constructs; therefore, MICE does not remove selection introduced by those exclusions. We created five imputed datasets, estimated each regression separately, and combined coefficients and standard errors according to Rubin’s rules. All analyses used R 4.4.3 and two-sided statistical tests with *p* < 0.05. To address multiplicity in the exploratory analyses, Benjamini-Hochberg FDR correction was applied separately within each outcome for subgroup tests in Supplementary Tables S6–S8 and across the eight overall interaction tests in each interaction parameterization (Supplementary Tables S9 and S10). Primary analyses were prespecified and were not multiplicity-adjusted. For nominal *p* values reported as <0.001 in the Supplementary Tables, 0.001 was used conservatively for the FDR calculation.

## 3. Results

### 3.1. Participant Characteristics

The final sample comprised 9278 participants. Comparison with excluded respondents is provided in Supplementary Table S1. LCA assigned 6404 participants (69.0%) to low SES, 940 (10.1%) to middle SES, and 1934 (20.9%) to high SES. The middle class was distinguished mainly by secondary education (57.30%), formal employment history (67.90%), and middle household income (56.52%). In the low class, 59.49% had no formal schooling and 96.81% had an informal occupation, whereas the high class showed greater educational attainment together with 98.03% formal occupation and 85.41% high household income (Supplementary Tables S2 and S3).

Participant profiles varied markedly across SES categories (Table 1). Relative to the high-SES class, the low-SES class contained more people aged ≥80 years (60.18% vs. 53.83%), more women (60.87% vs. 39.56%), more rural residents (53.53% vs. 8.07%), and more unmarried participants (58.45% vs. 40.62%). Mean CMMSE was lower in low SES (25.81 vs. 27.38), whereas mean social-isolation score was higher (2.85 vs. 2.29); mean ADL disability differed little (0.46 vs. 0.53). Depressive symptoms occurred in 52.30% of the low-SES class and 39.87% of the high-SES class; the corresponding percentages for anxiety symptoms were 13.66% and 8.01%. Correlations among the four diet scores were high and positive (r = 0.74–0.86; Supplementary Figure S1).

### 3.2. Associations of Socioeconomic Status with Depressive and Anxiety Symptoms

A socioeconomic gradient was evident for both outcomes (Table 2). After full adjustment, low SES versus high SES corresponded to an OR of 1.50 (95% CI: 1.32–1.71) for depressive symptoms, while the middle-SES estimate was 1.20 (95% CI: 1.01–1.41). For anxiety symptoms, the Model 3 ORs were 1.76 (95% CI: 1.43–2.16) for low SES and 1.43 (95% CI: 1.09–1.87) for middle SES. The elevated estimate for low SES persisted across the successive adjustment models.

### 3.3. Associations of Dietary Quality with Depressive and Anxiety Symptoms

All four diet-quality measures showed inverse associations with both symptom outcomes (Table 3). In Model 3, comparing participants above versus below the median yielded ORs for depressive symptoms of 0.64 (95% CI: 0.59–0.71) for cMIND, 0.60 (95% CI: 0.55–0.66) for HEI, 0.62 (95% CI: 0.57–0.68) for DDS, and 0.58 (95% CI: 0.52–0.64) for AIDDS. The anxiety estimates followed the same direction, ranging from 0.55 for AIDDS to 0.63 for cMIND.

On the continuous scale, a one-point increase in cMIND, HEI, DDS, and AIDDS corresponded to 15%, 4%, 14%, and 23% lower odds of depressive symptoms, respectively. The analogous reductions for anxiety symptoms were 14%, 4%, 14%, and 24% (all fully adjusted *p* < 0.001). Quartile models showed the same inverse pattern, with the highest quartile below the lowest for every index and *p* for trend < 0.001 (Supplementary Table S4).

Spline models indicated departures from linearity for cMIND (*p* nonlinearity = 0.023) and DDS (*p* nonlinearity = 0.012) with depressive symptoms, but not for HEI (*p* = 0.533) or AIDDS (*p* = 0.111). For anxiety symptoms, the nonlinearity tests were 0.157 for cMIND, 0.835 for HEI, 0.106 for DDS, and 0.011 for AIDDS, indicating evidence of a nonlinear AIDDS association only. Every spline model showed an overall association with its outcome (all *p* < 0.001; Supplementary Figure S2).

### 3.4. Joint Associations of Socioeconomic Status and Dietary Quality with Depressive and Anxiety Symptoms

The combined SES-diet categories showed a similar ordering across all four dietary measures (Table 4), with the least favorable estimates generally occurring when low SES coincided with low diet quality. Relative to high SES plus high cMIND, the low-SES/low-cMIND group had an OR of 1.97 (95% CI: 1.70–2.29) for depressive symptoms and 2.17 (95% CI: 1.71–2.75) for anxiety symptoms. HEI and DDS showed comparable joint patterns.

AIDDS produced the largest joint estimates. Compared with high SES plus high AIDDS, low SES plus low AIDDS was associated with ORs of 2.26 (95% CI: 1.94–2.64) for depressive symptoms and 2.69 (95% CI: 2.12–3.40) for anxiety symptoms. Within low SES, the estimates were generally more favorable for participants with higher diet quality than for those with lower diet quality, although these within-class contrasts were not the prespecified primary comparisons.

Accordingly, the joint categories are best viewed as identifying a subgroup in whom socioeconomic and dietary disadvantage co-occur with a particularly high symptom burden. They do not, by themselves, demonstrate interaction; SES-by-diet interaction terms were therefore evaluated in separate models.

### 3.5. Multiplicative Interaction Analyses

For median-dichotomized diet scores, the overall SES-by-diet interaction tests were not significant for either outcome (Supplementary Table S9). For depressive symptoms, the overall interaction *p* values were 0.395 (cMIND), 0.340 (HEI), 0.147 (DDS), and 0.363 (AIDDS); the corresponding values for anxiety symptoms were 0.074, 0.964, 0.719, and 0.649. After FDR correction across the eight overall interaction tests, none met q < 0.05 (all q ≥ 0.588).

Using standardized continuous diet scores, nominal overall interaction evidence was present for HEI (*p* = 0.041), DDS (*p* < 0.001), and AIDDS (*p* = 0.005), but not cMIND (*p* = 0.465) (Supplementary Table S10). After FDR correction across the eight overall tests, evidence remained for DDS (q ≤ 0.008) and AIDDS (q = 0.020), whereas HEI no longer met the FDR threshold (q = 0.109) and cMIND remained null (q = 0.685). No overall continuous-score interaction was evident for anxiety after correction (all q ≥ 0.685). These FDR-supported depression interactions remained exploratory and hypothesis-generating.

### 3.6. Sensitivity and Subgroup Analyses

Restricting the analysis to 5421 complete cases yielded SES, diet, and joint-exposure estimates that were broadly similar to those from multiple imputation (Supplementary Table S5). Results stratified by age and by sex generally retained the same direction, and the low-SES/low-diet groups usually showed the least favorable estimates within strata (Supplementary Tables S6–S8). Benjamini-Hochberg FDR correction did not change any nominally significant findings in the SES-only or continuous-diet subgroup analyses (Supplementary Tables S6 and S7). In the larger joint-exposure subgroup analysis (Supplementary Table S8), several borderline nominal associations no longer met q < 0.05, whereas the stronger low-SES/low-diet contrasts remained robust.

## 4. Discussion

This analysis of 9278 Chinese adults aged 65 years or older identified two parallel gradients in mental-health symptoms: lower SES and poorer diet quality were each associated with less favorable outcomes. When the exposures were considered jointly, the low-SES/low-diet categories consistently carried the largest estimates, most notably for AIDDS. The interaction analyses, however, qualify the interpretation of these joint categories. Median-dichotomized diet scores showed no overall interaction with SES. For standardized continuous scores, nominal interaction signals were seen for HEI, DDS, and AIDDS for depressive symptoms, but after FDR correction only DDS and AIDDS remained below q < 0.05, with no corrected interaction evidence for anxiety. The central message is therefore co-occurrence of social and dietary disadvantage, not consistent evidence of multiplicative synergy.

The SES gradient accords with evidence from late-life mental-health research [10,11,12,14,15,38]. A recent analysis spanning five prospective aging cohorts reported a 41% higher pooled risk of depressive symptoms for low SES and found the least favorable risk profile when low SES was accompanied by an unhealthy lifestyle (HR = 2.47) [12]. Our cross-sectional OR of 1.50 for low versus high SES points in the same direction but should not be numerically equated with a prospective hazard ratio. The corresponding anxiety estimate was stronger (OR = 1.76), consistent with reports of socioeconomic inequality in anxiety among older populations [11,15]. Financial strain, chronic psychosocial stress, fewer social and material resources, lower health literacy, restricted access to preventive care, and clustering of adverse health behaviors and chronic disease are plausible contributors [38,39,40,41,42,43]. In later life, retirement, multimorbidity, and contraction of social networks may further reduce the resources available to cope with these stressors.

Our diet results can be placed alongside several prior CLHLS studies. Inverse associations have been reported for cMIND with depressive and anxiety symptoms [19], cMIND and anti-inflammatory dietary diversity with anxiety [18], and HEI with depression [20] and anxiety [23]. In our data, the per-point HEI OR was 0.96 for both outcomes, somewhat stronger than the 0.97 and 0.977 estimates reported previously [20,23]; differences in sampling, covariate adjustment, missing-data procedures, and score specification make direct numerical comparison inappropriate. The shape of dose-response curves also varied. Niu et al. described an approximately linear cMIND relation with both outcomes [19], whereas our spline suggested nonlinearity for depressive symptoms. Conversely, earlier HEI studies reported nonlinear associations [20,23], while our HEI curves were compatible with linearity. Such discrepancies reinforce that spline shape is sensitive to the analytic sample and model specification, whereas the direction of the overall diet associations was comparatively stable.

The four dietary measures should not be regarded as independent replications. They came from the same abbreviated FFQ, share many component foods, and were strongly correlated (r = 0.74–0.86). Their concordance therefore provides a sensitivity check across overlapping representations of dietary quality rather than four separate evidence streams. The comparative value lies in showing that differently weighted summaries of the same underlying dietary information led to a similar inverse pattern.

The joint findings also fit the broader observation that socioeconomic disadvantage and adverse health behaviors tend to cluster. In another CLHLS analysis, the combination of sufficient dietary diversity and current exercise corresponded to substantially lower odds of depressive symptoms, anxiety symptoms, and their co-occurrence than less favorable behavior profiles [26]. The five-cohort study likewise reported the greatest depression risk when low SES was accompanied by an unfavorable lifestyle [12]. Although the exposures and effect measures differ from ours, both studies support examining combined risk profiles. Importantly, our formal tests show that a large contrast between joint categories does not establish statistical or biological interaction: none of the dichotomized diet measures showed an overall multiplicative interaction with SES, before or after FDR correction. For continuous scores, the nominal HEI interaction did not survive FDR correction, whereas DDS and AIDDS remained below q < 0.05 for depressive symptoms. These corrected findings were absent for anxiety and remain exploratory, requiring confirmation in independent data.

Several pathways could connect socioeconomic position, diet, and mental health. More favorable dietary patterns can supply micronutrients, fiber, and bioactive compounds relevant to neurotransmitter production, gut-microbiome function, metabolic regulation, oxidative stress, and inflammation [16,44,45]. At the same time, socioeconomic disadvantage may increase chronic stress while narrowing access to affordable and diverse nutrient-dense foods [13,24,25]. These mechanisms make co-occurrence plausible, but the present data cannot determine mediation or demonstrate that changing diet would offset socioeconomic disadvantage. The clinical and public-health implication is therefore one of identifying a high-burden group: older adults facing both social disadvantage and poor diet quality may warrant integrated nutritional, social, and mental-health support.

Several features strengthen the analysis: the large community-based sample; a multidimensional SES measure derived jointly from education, income, and pre-60 occupation; parallel evaluation of four dietary scores and two mental-health outcomes; and a series of complementary analyses using imputation, complete cases, quartiles, splines, stratification, joint categories, and formal interaction terms.

The findings also require several qualifications. First, the cross-sectional design cannot establish the temporal ordering of SES, diet, and symptoms. Reverse causation is plausible because depression or anxiety may change appetite, food selection, or how dietary behavior is reported. Second, 6596 of the 15,874 initial respondents were not included in the analytic cohort, mainly because SES indicators or mental-health assessments were incomplete. As shown in Supplementary Table S1, excluded respondents were older, more often women and unmarried, and had less favorable socioeconomic, cognitive, functional, and social-isolation profiles. The resulting sample may therefore underrepresent frailer and more socially disadvantaged older adults, with potential consequences for both selection bias and generalizability. Because MICE was restricted to missing covariates after analytic eligibility was established, it could not recover participants excluded for missing SES indicators, diet data, or symptom assessments. Third, the abbreviated self-reported FFQ records broad intake-frequency categories but not portion size, total energy intake, or detailed nutrient composition and is vulnerable to recall and classification error.

Fourth, CES-D-10 and GAD-7 are screening scales for symptom burden, not diagnostic interviews. The cut points used here include mild symptoms and should not be interpreted as diagnoses of major depressive disorder or generalized anxiety disorder; geriatric-specific instruments such as the Geriatric Depression Scale and Geriatric Anxiety Scale were unavailable in the CLHLS. Fifth, residual confounding remains possible despite additional adjustment for cognition, ADL disability, social isolation, and chronic disease. A formal frailty measure was not included, and detailed information was insufficient for disease severity, painful conditions, bereavement, specific psychotropic and other medication classes, physician-prescribed diets, and access to healthcare, all of which could influence both eating behavior and mental-health symptoms. Chronic disease may also occupy part of the causal pathway, so Model 3 represents an adjusted association rather than a direct causal effect. Sixth, the SES construct did not include wealth, neighborhood deprivation, or perceived social rank, and LCA class definitions are sample dependent. Finally, although Benjamini-Hochberg FDR correction was applied to the exploratory subgroup and interaction analyses, the large number of exploratory contrasts and their data-dependent interpretation still warrant caution; we therefore emphasize corrected q values, overall interaction tests, and reproducible patterns rather than isolated nominal *p* values.

## 5. Conclusions

In the 2018 CLHLS, socioeconomic disadvantage and poorer diet quality were each associated with more depressive and anxiety symptoms in older adults, and their combination identified the least favorable joint profiles. Similar patterns appeared across four correlated dietary indices and multiple robustness analyses. The dichotomized diet measures did not show a general multiplicative interaction with SES, and after FDR correction the exploratory continuous-score interaction signals remained for DDS and AIDDS, but not HEI, for depressive symptoms. These observations support considering social circumstances, diet, and mental health together when identifying vulnerable older adults, while causal claims about dietary improvement and SES-related mental-health inequalities await longitudinal and intervention studies.

## Figures and Tables

**Table 1:** Characteristics of the 2018 Chinese Longitudinal Healthy Longevity Survey analytic sample according to socioeconomic status.

Characteristics		Overall (*n* = 9278)	SES			*p* Value
			Low (*n* = 6404)	Middle (*n* = 940)	High (*n* = 1934)	
**Age (years)**	65–79	3870 (41.71)	2550 (39.82)	427 (45.43)	893 (46.17)	<0.001
	≥80	5408 (58.29)	3854 (60.18)	513 (54.57)	1041 (53.83)	
**Sex**	Male	4253 (45.84)	2506 (39.13)	578 (61.49)	1169 (60.44)	<0.001
	Female	5025 (54.16)	3898 (60.87)	362 (38.51)	765 (39.56)	
Residence	Urban	5466 (58.91)	2976 (46.47)	712 (75.74)	1778 (91.93)	<0.001
	Rural	3812 (41.09)	3428 (53.53)	228 (24.26)	156 (8.07)	
Marital status *	Married and living with spouse	4216 (45.78)	2640 (41.55)	433 (46.56)	1143 (59.38)	<0.001
	Unmarried	4993 (54.22)	3714 (58.45)	497 (53.44)	782 (40.62)	
Smoking *	Never	6278 (68.63)	4474 (70.86)	537 (57.80)	1267 (66.54)	<0.001
	Former	1392 (15.22)	814 (12.89)	193 (20.78)	385 (20.22)	
	Current	1477 (16.15)	1026 (16.25)	199 (21.42)	252 (13.24)	
Drinking *	Never	6650 (72.92)	4670 (74.16)	624 (67.53)	1356 (71.41)	<0.001
	Former	1077 (11.81)	717 (11.39)	119 (12.88)	241 (12.69)	
	Current	1393 (15.27)	910 (14.45)	181 (19.59)	302 (15.90)	
Exercise *	Never	5171 (56.46)	4179 (66.17)	357 (38.47)	635 (33.16)	<0.001
	Former	726 (7.93)	348 (5.51)	112 (12.07)	266 (13.89)	
	Current	3262 (35.62)	1789 (28.32)	459 (49.46)	1014 (52.95)	
BMI *	<18.5	1016 (11.88)	811 (13.85)	68 (7.79)	137 (7.52)	<0.001
	18.5–<24.0	4563 (53.36)	3236 (55.25)	455 (52.12)	872 (47.86)	
	≥24.0	2973 (34.76)	1810 (30.90)	350 (40.09)	813 (44.62)	
Hypertension *	No	4868 (55.22)	3646 (60.21)	439 (48.56)	783 (42.16)	<0.001
	Yes	3948 (44.78)	2409 (39.79)	465 (51.44)	1074 (57.84)	
Diabetes *	No	7491 (88.11)	5408 (92.35)	729 (82.84)	1354 (76.67)	<0.001
	Yes	1011 (11.89)	448 (7.65)	151 (17.16)	412 (23.33)	
Heart disease *	No	6920 (81.13)	5050 (86.03)	676 (76.64)	1194 (67.19)	<0.001
	Yes	1609 (18.87)	820 (13.97)	206 (23.36)	583 (32.81)	
Stroke or other cerebrovascular disease *	No	7456 (88.09)	5316 (91.04)	749 (86.09)	1391 (79.26)	<0.001
	Yes	1008 (11.91)	523 (8.96)	121 (13.91)	364 (20.74)	
Respiratory disease *	No	7434 (89.31)	5197 (90.43)	758 (89.07)	1479 (85.69)	<0.001
	Yes	890 (10.69)	550 (9.57)	93 (10.93)	247 (14.31)	
Depressive symptoms	No (CES-D score < 10)	4751 (51.21)	3055 (47.70)	533 (56.70)	1163 (60.13)	<0.001
	Yes (CES-D score ≥ 10)	4527 (48.79)	3349 (52.30)	407 (43.30)	771 (39.87)	
Anxiety symptoms	No (GAD-7 score < 5)	8149 (87.83)	5529 (86.34)	841 (89.47)	1779 (91.99)	<0.001
	Yes (GAD-7 score ≥ 5)	1129 (12.17)	875 (13.66)	99 (10.53)	155 (8.01)	
CMMSE score * (mean (SD))		26.37 (5.46)	25.81 (5.84)	27.10 (4.87)	27.38 (4.49)	<0.001
ADL disability score * (mean (SD))		0.48 (1.25)	0.46 (1.21)	0.55 (1.36)	0.53 (1.32)	0.027
isolation score * (mean (SD))		2.71 (1.11)	2.85 (1.07)	2.65 (1.14)	2.29 (1.13)	<0.001

Notes: Categorical variables are shown as *n* (%) and continuous variables as mean (SD). For variables with missing observations, percentages and means use the available denominator. Between-group *p* values came from chi-square tests for categorical variables and one-way analysis of variance for continuous variables. * Missing counts were: marital status, 69; smoking, 131; drinking, 158; exercise, 119; BMI, 726; hypertension, 462; diabetes, 776; heart disease, 749; stroke/other cerebrovascular disease, 814; respiratory disease, 954; CMMSE, 2445; ADL disability, 50; and social isolation, 24. Abbreviations: ADL, activities of daily living; BMI, body mass index; CES-D, Center for Epidemiologic Studies Depression Scale; CMMSE, Chinese version of the Mini-Mental State Examination; GAD-7, 7-item Generalized Anxiety Disorder Scale; SD, standard deviation; SES, socioeconomic status.

**Table 2:** Socioeconomic-status associations with depressive and anxiety symptoms in the 2018 Chinese Longitudinal Healthy Longevity Survey.

SES	Model 1		Model 2		Model 3	
	OR (95% CI)	*p* Value	OR (95% CI)	*p* Value	OR (95% CI)	*p* Value
**Depressive symptoms**						
High SES	Ref.		Ref.		Ref.	
Middle SES	1.15 (0.98–1.35)	0.079	1.12 (0.95–1.31)	0.169	1.20 (1.01–1.41)	0.037
Low SES	1.65 (1.49–1.83)	<0.001	1.52 (1.36–1.70)	<0.001	1.50 (1.32–1.71)	<0.001
**Anxiety symptoms**						
High SES	Ref.		Ref.		Ref.	
Middle SES	1.35 (1.04–1.76)	0.026	1.36 (1.04–1.77)	0.026	1.43 (1.09–1.87)	0.011
Low SES	1.82 (1.52–2.17)	<0.001	1.72 (1.42–2.08)	<0.001	1.76 (1.43–2.16)	<0.001

Notes: Model 1 contained no covariates. Model 2 included age, sex, residence, and marital status. Model 3 additionally included smoking, drinking, exercise, BMI, hypertension, diabetes, heart disease, stroke or other cerebrovascular disease, respiratory disease, CMMSE, ADL disability, and social isolation. Results from the five imputed datasets were combined with Rubin’s rules. Abbreviations: ADL, activities of daily living; BMI, body mass index; CI, confidence interval; CMMSE, Chinese version of the Mini-Mental State Examination; OR, odds ratio; SES, socioeconomic status.

**Table 3:** Diet-quality scores in relation to depressive and anxiety symptoms in the 2018 Chinese Longitudinal Healthy Longevity Survey.

Dietary Score	Model 1		Model 2		Model 3	
	OR (95% CI)	*p* Value	OR (95% CI)	*p* Value	OR (95% CI)	*p* Value
**Depressive symptoms**						
**cMIND**						
High (ref: low)	0.53 (0.49–0.58)	<0.001	0.58 (0.53–0.63)	<0.001	0.64 (0.59–0.71)	<0.001
Continuous (per 1-point increment)	0.80 (0.78–0.82)	<0.001	0.82 (0.80–0.84)	<0.001	0.85 (0.83–0.87)	<0.001
**HEI**						
High (ref: low)	0.52 (0.48–0.56)	<0.001	0.55 (0.50–0.60)	<0.001	0.60 (0.55–0.66)	<0.001
Continuous (per 1-point increment)	0.95 (0.94–0.95)	<0.001	0.95 (0.94–0.96)	<0.001	0.96 (0.95–0.96)	<0.001
**DDS**						
High (ref: low)	0.54 (0.49–0.58)	<0.001	0.57 (0.52–0.62)	<0.001	0.62 (0.57–0.68)	<0.001
Continuous (per 1-point increment)	0.83 (0.82–0.85)	<0.001	0.84 (0.83–0.86)	<0.001	0.86 (0.84–0.88)	<0.001
**AIDDS**						
High (ref: low)	0.48 (0.43–0.53)	<0.001	0.51 (0.46–0.56)	<0.001	0.58 (0.52–0.64)	<0.001
Continuous (per 1-point increment)	0.71 (0.69–0.74)	<0.001	0.73 (0.71–0.76)	<0.001	0.77 (0.74–0.80)	<0.001
**Anxiety symptoms**						
**cMIND**						
High (ref: low)	0.57 (0.51–0.65)	<0.001	0.58 (0.51–0.66)	<0.001	0.63 (0.55–0.72)	<0.001
Continuous (per 1-point increment)	0.84 (0.81–0.87)	<0.001	0.84 (0.81–0.87)	<0.001	0.86 (0.83–0.89)	<0.001
**HEI**						
High (ref: low)	0.56 (0.49–0.63)	<0.001	0.57 (0.50–0.65)	<0.001	0.61 (0.53–0.70)	<0.001
Continuous (per 1-point increment)	0.95 (0.94–0.96)	<0.001	0.95 (0.94–0.96)	<0.001	0.96 (0.95–0.97)	<0.001
**DDS**						
High (ref: low)	0.55 (0.48–0.62)	<0.001	0.56 (0.50–0.64)	<0.001	0.60 (0.52–0.68)	<0.001
Continuous (per 1-point increment)	0.84 (0.81–0.87)	<0.001	0.84 (0.82–0.87)	<0.001	0.86 (0.83–0.89)	<0.001
**AIDDS**						
High (ref: low)	0.48 (0.42–0.55)	<0.001	0.49 (0.43–0.56)	<0.001	0.55 (0.47–0.63)	<0.001
Continuous (per 1-point increment)	0.73 (0.69–0.77)	<0.001	0.73 (0.69–0.77)	<0.001	0.76 (0.72–0.81)	<0.001

Notes: For categorical analyses, high and low diet quality were separated at the median of each score within each imputed dataset. Continuous ORs correspond to a one-point increase in the named score. Model 1 had no covariates; Model 2 included age, sex, residence, and marital status; and Model 3 further included smoking, drinking, exercise, BMI, hypertension, diabetes, heart disease, stroke or other cerebrovascular disease, respiratory disease, CMMSE, ADL disability, and social isolation. Estimates across the five imputed datasets were combined using Rubin’s rules. Abbreviations: ADL, activities of daily living; AIDDS, Anti-Inflammatory Dietary Diversity Score; BMI, body mass index; CI, confidence interval; cMIND, Chinese version of the Mediterranean-DASH Intervention for Neurodegenerative Delay; CMMSE, Chinese version of the Mini-Mental State Examination; DDS, Dietary Diversity Score; HEI, Healthy Eating Index; OR, odds ratio.

**Table 4:** Joint socioeconomic-status and diet-quality categories in relation to depressive and anxiety symptoms in the 2018 Chinese Longitudinal Healthy Longevity Survey.

Joint Exposure Group	Depressive Symptoms		Anxiety Symptoms	
	OR (95% CI)	*p* Value	OR (95% CI)	*p* Value
**cMIND + SES**				
High diet + High SES	Ref.		Ref.	
High diet + Middle SES	1.08 (0.89–1.32)	0.423	1.12 (0.80–1.58)	0.511
High diet + Low SES	1.31 (1.13–1.52)	<0.001	1.42 (1.12–1.81)	0.004
Low diet + High SES	1.30 (1.02–1.65)	0.031	1.01 (0.67–1.52)	0.959
Low diet + Middle SES	1.80 (1.38–2.35)	<0.001	2.21 (1.51–3.23)	<0.001
Low diet + Low SES	1.97 (1.70–2.29)	<0.001	2.17 (1.71–2.75)	<0.001
**HEI + SES**				
High diet + High SES	Ref.		Ref.	
High diet + Middle SES	1.08 (0.89–1.31)	0.433	1.35 (0.98–1.86)	0.070
High diet + Low SES	1.25 (1.08–1.44)	0.002	1.55 (1.22–1.96)	<0.001
Low diet + High SES	1.32 (1.00–1.74)	0.048	1.61 (1.05–2.48)	0.031
Low diet + Middle SES	1.85 (1.40–2.44)	<0.001	2.14 (1.43–3.22)	<0.001
Low diet + Low SES	2.03 (1.76–2.35)	<0.001	2.36 (1.86–2.98)	<0.001
**DDS + SES**				
High diet + High SES	Ref.		Ref.	
High diet + Middle SES	1.15 (0.95–1.39)	0.164	1.45 (1.05–2.00)	0.024
High diet + Low SES	1.25 (1.08–1.44)	0.002	1.53 (1.20–1.95)	<0.001
Low diet + High SES	1.27 (0.99–1.65)	0.065	1.65 (1.10–2.47)	0.014
Low diet + Middle SES	1.56 (1.18–2.05)	0.002	1.92 (1.27–2.91)	0.002
Low diet + Low SES	2.03 (1.75–2.35)	<0.001	2.44 (1.93–3.09)	<0.001
**AIDDS + SES**				
High diet + High SES	Ref.		Ref.	
High diet + Middle SES	1.11 (0.93–1.33)	0.241	1.34 (0.99–1.80)	0.059
High diet + Low SES	1.35 (1.18–1.54)	<0.001	1.51 (1.21–1.88)	<0.001
Low diet + High SES	1.34 (0.94–1.89)	0.101	1.37 (0.79–2.35)	0.261
Low diet + Middle SES	2.11 (1.47–3.03)	<0.001	2.28 (1.40–3.72)	<0.001
Low diet + Low SES	2.26 (1.94–2.64)	<0.001	2.69 (2.12–3.40)	<0.001

Notes: Each diet score was split at its imputation-specific median and crossed with the three SES classes, yielding six categories. The reference was high SES with high diet quality for the relevant index. Models included age, sex, residence, marital status, smoking, drinking, exercise, BMI, hypertension, diabetes, heart disease, stroke or other cerebrovascular disease, respiratory disease, CMMSE, ADL disability, and social isolation. The five imputation-specific estimates were combined with Rubin’s rules. Abbreviations: ADL, activities of daily living; AIDDS, Anti-Inflammatory Dietary Diversity Score; BMI, body mass index; CI, confidence interval; cMIND, Chinese version of the Mediterranean-DASH Intervention for Neurodegenerative Delay; CMMSE, Chinese version of the Mini-Mental State Examination; DDS, Dietary Diversity Score; HEI, Healthy Eating Index; OR, odds ratio; SES, socioeconomic status.

## Data Availability

The CLHLS data used in this study are available through the Peking University Open Research Data Platform http://opendata.pku.edu.cn/ (accessed on 25 September 2026), subject to the data provider’s access procedures.

## Supplementary Materials

The following supporting information can be downloaded at: https://www.mdpi.com/article/10.3390/nu18213540/s1, Supplementary Table S1. Baseline characteristics of included and excluded participants in the 2018 wave of the Chinese Longitudinal Healthy Longevity Survey. Supplementary Table S2. Model-fit indices for latent class analysis of socioeconomic status. Supplementary Table S3. Indicator distributions across the three latent socioeconomic status classes. Supplementary Table S4. Associations of quartiles of dietary quality scores with depressive and anxiety symptoms in the 2018 wave of the Chinese Longitudinal Healthy Longevity Survey. Supplementary Table S5. Complete-case sensitivity analyses of socioeconomic status, dietary quality, and their joint associations with depressive and anxiety symptoms in the 2018 wave of the Chinese Longitudinal Healthy Longevity Survey (*n* = 5421). Supplementary Table S6. Subgroup analyses of socioeconomic status with depressive and anxiety symptoms. Supplementary Table S7. Subgroup analyses of dietary quality scores with depressive and anxiety symptoms. Supplementary Table S8. Subgroup analyses of joint socioeconomic status-dietary quality associations with depressive and anxiety symptoms. Supplementary Table S9. Multiplicative interactions between socioeconomic status and dichotomized dietary quality in relation to depressive and anxiety symptoms. Supplementary Table S10. Multiplicative interactions between socioeconomic status and standardized continuous dietary quality scores in relation to depressive and anxiety symptoms. Supplementary Figure S1. Correlation matrix of the four dietary quality indices in the 2018 wave of the Chinese Longitudinal Healthy Longevity Survey. Supplementary Figure S2. Restricted cubic spline associations of the four dietary quality indices with depressive and anxiety symptoms in the 2018 wave of the Chinese Longitudinal Healthy Longevity Survey. Panels A–D show depressive symptoms for cMIND, HEI, DDS, and AIDDS, respectively; panels E–H show anxiety symptoms for the corresponding dietary indices.

## Abbreviations

The following abbreviations are used in this manuscript:

CLHLS	Chinese Longitudinal Healthy Longevity Survey
LCA	latent class analysis
SES	socioeconomic status
cMIND	Chinese version of the Mediterranean-DASH Intervention for Neurodegenerative Delay
HEI	Healthy Eating Index
DDS	Dietary Diversity Score
AIDDS	Anti-Inflammatory Dietary Diversity Score
CES-D-10	10-item Center for Epidemiologic Studies Depression Scale
GAD-7	7-item Generalized Anxiety Disorder Scale
ADL	activities of daily living
CMMSE	the Chinese version of the Mini-Mental State Examination
ANOVA	one-way analysis of variance
OR	odds ratio
CI	confidence interval
AIC	Akaike information criterion
BIC	Bayesian information criterion
FFQ	food-frequency questionnaire
BMI	body mass index
MICE	multiple imputation by chained equations
RCS	restricted cubic spline
SD	standard deviation
