# Supplementary material for: Independent and Joint Associations of Socioeconomic Status and Dietary Quality with Depressive and Anxiety Symptoms Among Chinese Older Adults: A Cross-Sectional Study of the Chinese Longitudinal Healthy Longevity Survey
Source: Nutrients. 2026 Oct 27;18(21):3540. doi: 10.3390/nu18213540 (PMC13765185; doi:10.3390/nu18213540)
Supplement: Supplementary file 1 [file nutrients-18-03540-s001.zip › Supplementary Figure S1.pdf]

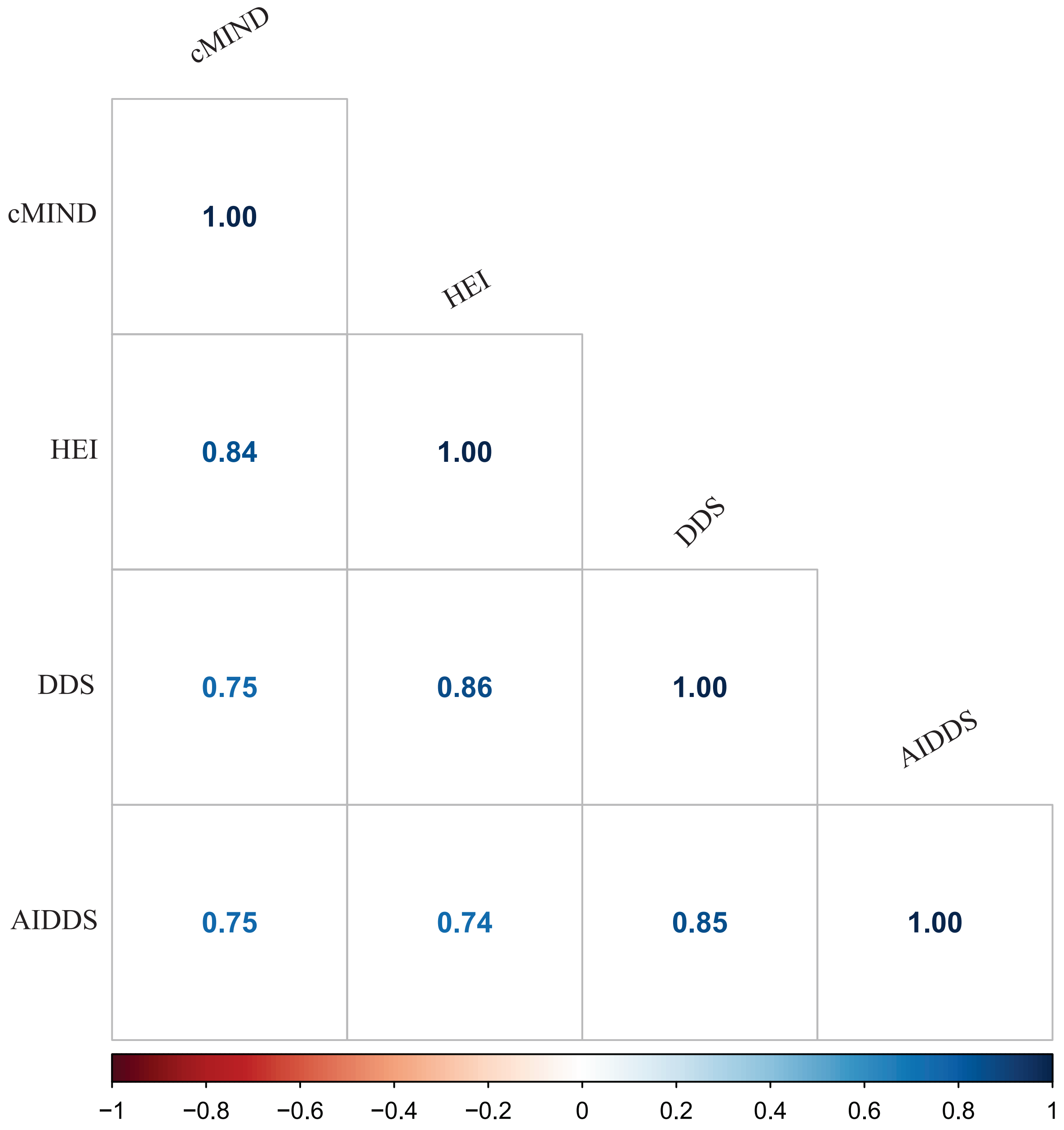
cMIND
HEI
DDS
AIDDS
1.00
0.84
1.00
0.75
0.86
1.00
0.75
0.74
0.85
1.00
−1
−0.8
−0.6
−0.4
−0.2
0
0.2
0.4
0.6
0.8
1
