# Supplementary material for: Independent and Joint Associations of Socioeconomic Status and Dietary Quality with Depressive and Anxiety Symptoms Among Chinese Older Adults: A Cross-Sectional Study of the Chinese Longitudinal Healthy Longevity Survey
Source: Nutrients. 2026 Oct 27;18(21):3540. doi: 10.3390/nu18213540 (PMC13765185; doi:10.3390/nu18213540)
Supplement: Supplementary file 1 [file nutrients-18-03540-s001.zip › Supplementary Figure S2.pdf]

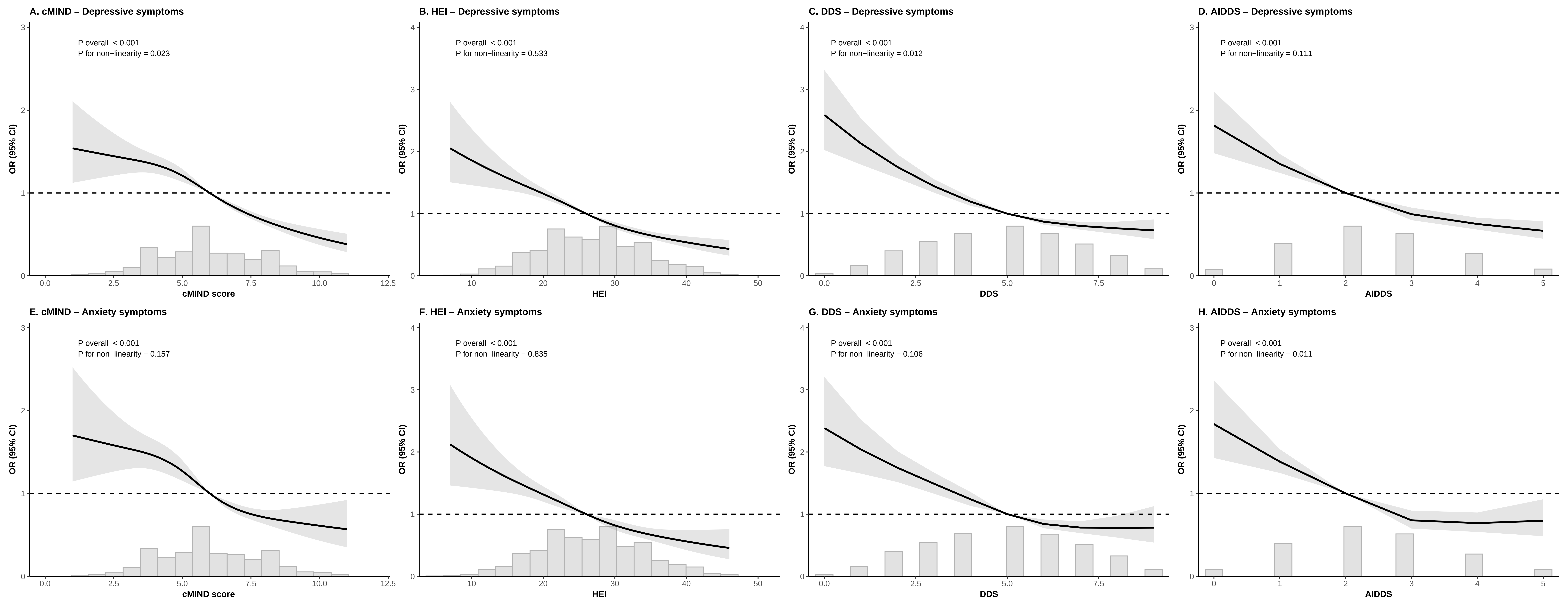
A. cMIND – Depressive symptoms
P overall < 0.001
P for non−linearity = 0.023
OR (95% CI)
cMIND score
B. HEI – Depressive symptoms
P overall < 0.001
P for non−linearity = 0.533
OR (95% CI)
HEI
C. DDS – Depressive symptoms
P overall < 0.001
P for non−linearity = 0.012
OR (95% CI)
DDS
D. AIDDS – Depressive symptoms
P overall < 0.001
P for non−linearity = 0.111
OR (95% CI)
AIDDS
E. cMIND – Anxiety symptoms
P overall < 0.001
P for non−linearity = 0.157
OR (95% CI)
cMIND score
F. HEI – Anxiety symptoms
P overall < 0.001
P for non−linearity = 0.835
OR (95% CI)
HEI
G. DDS – Anxiety symptoms
P overall < 0.001
P for non−linearity = 0.106
OR (95% CI)
DDS
H. AIDDS – Anxiety symptoms
P overall < 0.001
P for non−linearity = 0.011
OR (95% CI)
AIDDS
