# Supplementary material for: Independent and Joint Associations of Socioeconomic Status and Dietary Quality with Depressive and Anxiety Symptoms Among Chinese Older Adults: A Cross-Sectional Study of the Chinese Longitudinal Healthy Longevity Survey
Source: Nutrients. 2026 Oct 27;18(21):3540. doi: 10.3390/nu18213540 (PMC13765185; doi:10.3390/nu18213540)
Supplement: Supplementary file 1 [file nutrients-18-03540-s001.zip › Supplementary Tables S1-S10.pdf]

Supplementary Table S1. Baseline Characteristics of included and excluded participants in the 2018 wave of the Chinese Longitudinal Healthy Longevity Survey.

Characteristics		Overall (n = 15,874)	Excluded (n = 6,596)	Included (n = 9,278)	P value
Age* (years)	< 80	5447 (34.33)	1577 (23.94)	3870 (41.71)	<0.001
	≥ 80	10419 (65.67)	5011 (76.06)	5408 (58.29)	
Sex	Male	6925 (43.62)	2672 (40.51)	4253 (45.84)	<0.001
	Female	8949 (56.38)	3924 (59.49)	5025 (54.16)	
Residence	Urban	8780 (55.31)	3314 (50.24)	5466 (58.91)	<0.001
	Rural	7094 (44.69)	3282 (49.76)	3812 (41.09)	
Marital status*	Married and living with spouse	6135 (39.31)	1919 (29.99)	4216 (45.78)	<0.001
	Unmarried	9472 (60.69)	4479 (70.01)	4993 (54.22)	
Smoking*	Never	10930 (70.45)	4652 (73.05)	6278 (68.63)	<0.001
	Former	2280 (14.70)	888 (13.94)	1392 (15.22)	
	Current	2305 (14.86)	828 (13.00)	1477 (16.15)	
Drinking*	Never	11471 (74.27)	4821 (76.22)	6650 (72.92)	<0.001
	Former	1782 (11.54)	705 (11.15)	1077 (11.81)	
	Current	2192 (14.19)	799 (12.63)	1393 (15.27)	
Exercise*	Never	9628 (62.20)	4457 (70.51)	5171 (56.46)	<0.001
	Former	1209 (7.81)	483 (7.64)	726 (7.93)	
	Current	4643 (29.99)	1381 (21.85)	3262 (35.62)	
Hypertension*	No	8781 (59.06)	3913 (64.66)	4868 (55.22)	<0.001
	Yes	6087 (40.94)	2139 (35.34)	3948 (44.78)	
Diabetes*	No	12957 (90.24)	5466 (93.32)	7491 (88.11)	<0.001
	Yes	1402 (9.76)	391 (6.68)	1011 (11.89)	
Heart disease*	No	11979 (83.10)	5059 (85.95)	6920 (81.13)	<0.001
	Yes	2436 (16.90)	827 (14.05)	1609 (18.87)	
Stroke or other cerebrovascular disease*	No	12712 (88.65)	5256 (89.45)	7456 (88.09)	0.013
	Yes	1628 (11.35)	620 (10.55)	1008 (11.91)	
Respiratory disease*	No	12633 (89.68)	5199 (90.23)	7434 (89.31)	0.082
	Yes	1453 (10.32)	563 (9.77)	890 (10.69)	
Education* (years)	0 years	6807 (50.41)	2802 (66.34)	4005 (43.17)	<0.001
	1-5 years	2994 (22.17)	791 (18.73)	2203 (23.74)	
	6-12 years	3229 (23.91)	563 (13.33)	2666 (28.73)	
	≥13 years	472 (3.50)	68 (1.61)	404 (4.35)	
Occupation*	formal	3755 (27.86)	802 (19.09)	2953 (31.83)	<0.001
	informal	9725 (72.14)	3400 (80.91)	6325 (68.17)	
Income*	Low income (≤10000 yuan)	4830 (33.66)	1877 (37.01)	2953 (31.83)	<0.001
	Middle income (>10000 ~ ≤50000 yuan)	4904 (34.18)	1840 (36.28)	3064 (33.02)	
	High income (>50000 yuan)	4615 (32.16)	1354 (26.70)	3261 (35.15)	

BMI* (mean (SD)) (kg/m^2)	22.50 (3.69)	22.09 (3.72)	22.75 (3.64)	<0.001
CMMSE score* (mean (SD))	23.93 (8.72)	19.69 (11.33)	26.37 (5.46)	<0.001
ADL disability score* (mean (SD))	0.81 (1.68)	1.28 (2.06)	0.48 (1.25)	<0.001
isolation score (mean (SD))	2.85 (1.08)	3.05 (1.00)	2.71 (1.11)	<0.001

Notes: Data are presented as n (%) for categorical variables and mean (SD) for continuous variables. Percentages for variables with missing data were calculated among participants with nonmissing observations. P values were calculated using chi-square tests for categorical variables and ANOVA for continuous variables. Variables with missing values were age (n = 8), marital status (n = 267), smoking (n = 359), drinking (n = 429), exercise (n = 394), BMI (n = 2121), hypertension (n = 1006), diabetes (n = 1515), heart disease (n = 1459), stroke or other cerebrovascular disease (n = 1534), respiratory disease (n = 1788), education (n = 2,372) , occupation (n = 2,394), income (n = 1,525), CMMSE score (n = 5111), ADL disability Score (n = 201), and the isolation score (n = 234). Abbreviations: ADL, activities of daily living; ANOVA, one-way analysis of variance; BMI, body mass index; CES-D, Center for Epidemiologic Studies Depression Scale; CMMSE, the Chinese version of the Mini-Mental State Examination; GAD-7, 7-item Generalized Anxiety Disorder Scale; SD, standard deviation; SES, socioeconomic status.

Supplementary Table S2. Model-fit indices for latent class analysis of socioeconomic status.

Number of classes	Log-likelihood	Number of parameters	AIC	BIC	G^2	χ^2	Class proportions, %	Mean posterior probability	Entropy	R-entropy
1	-27112.04	6	54236.08	54278.9	4415.41	5754.81				
2	-24952.87	13	49931.73	50024.49	97.06	90.02	28.73/71.27			
3	-24909.03	20	49858.06	50000.77	9.39	9.34	10.13/69.02/20.85	0.89	0.32	0.97
4	-24904.38	27	49862.76	50055.41	0.084	0.084	17.35/39.63/22.17/20.85			
5	-24904.34	34	49876.67	50119.28	<0.001	<0.001	8.17/4.11/14.70/20.85/52.18			

Notes: The three-class solution was selected for the primary analysis. Abbreviations: AIC, Akaike information criterion; BIC, Bayesian information criterion; G^2, likelihood-ratio chi-square statistic; LCA, latent class analysis; SES, socioeconomic status; χ^2, Pearson chi-square statistic.

Supplementary Table S3. Indicator distributions across the three latent socioeconomic status classes.

Variables	Proportion			
Education	No education (0 years)	Elementary school (1-5 years)	Secondary school (6-12 years)	University or above (≥13 years)
Low SES (class 2)	0.5949	0.2605	0.1430	0.0015
Middle SES (class 1)	0.1494	0.2776	0.5730	0.0000
High SES (class 3)	0.1031	0.1399	0.5548	0.2021
Occupation	Formal occupation	Informal occupation		
Low SES (class 2)	0.0319	0.9681		
Middle SES (class 1)	0.6790	0.3210		
High SES (class 3)	0.9803	0.0197		
Annual household income	Low income (≤10,000 yuan)	Middle income (>10,000 to ≤50,000 yuan)	High income (>50,000 yuan)	
Low SES (class 2)	0.4267	0.3579	0.2154	
Middle SES (class 1)	0.2070	0.5652	0.2278	
High SES (class 3)	0.0514	0.0945	0.8541	

Notes: Values are conditional proportions within each latent SES class. Abbreviation: SES, socioeconomic status.

Supplementary Table S4. Associations of quartiles of dietary quality scores with depressive and anxiety symptoms in the 2018 wave of the Chinese Longitudinal Healthy Longevity Survey.

Variables	OR (95% CI)				P for trend
	Q1	Q2	Q3	Q4	
**Depressive symptoms**					
cMIND	Ref.	0.79 (0.70–0.89)	0.68 (0.61–0.77)	0.45 (0.39–0.51)	<0.001
HEI	Ref.	0.74 (0.65–0.83)	0.56 (0.50–0.63)	0.46 (0.40–0.53)	<0.001
DDS	Ref.	0.66 (0.59–0.75)	0.58 (0.52–0.66)	0.47 (0.42–0.54)	<0.001
AIDDS	Ref.	0.71 (0.63–0.80)	0.59 (0.52–0.67)	0.48 (0.43–0.55)	<0.001
**Anxiety symptoms**					
cMIND	Ref.	0.79 (0.67–0.93)	0.64 (0.54–0.77)	0.48 (0.39–0.59)	<0.001
HEI	Ref.	0.89 (0.76–1.05)	0.62 (0.52–0.74)	0.51 (0.42–0.63)	<0.001
DDS	Ref.	0.71 (0.60–0.84)	0.59 (0.49–0.70)	0.44 (0.36–0.54)	<0.001
AIDDS	Ref.	0.66 (0.56–0.78)	0.55 (0.46–0.65)	0.46 (0.38–0.56)	<0.001

Notes: Q1 was the reference category. Models were adjusted for age, sex, residence, marital status, smoking, drinking, exercise, BMI, hypertension, diabetes, heart disease, stroke or other cerebrovascular disease, respiratory disease, CMMSE score, ADL disability Score, and isolation score (Model 3 covariates). Regression estimates were pooled across five multiply imputed datasets using Rubin's rules. Abbreviations: ADL, activities of daily living; AIDDS, Anti-Inflammatory Dietary Diversity Score; BMI, body mass index; CI, confidence interval; CLHLS, Chinese Longitudinal Healthy Longevity Survey; cMIND, Chinese version of the Mediterranean-DASH Intervention for Neurodegenerative Delay; CMMSE, the Chinese version of the Mini-Mental State Examination; DDS, Dietary Diversity Score; HEI, Healthy Eating Index; OR, odds ratio; Q, quartile.

Supplementary Table S5. Complete-case sensitivity analyses of socioeconomic status, dietary quality, and their joint associations with depressive and anxiety symptoms in the 2018 wave of the Chinese Longitudinal Healthy Longevity Survey (n = 5,421).

	Depressive symptoms		Anxiety symptoms	
	OR (95% CI)	P value	OR (95% CI)	P value
**Individual SES (ref: High SES)**				
Middle SES	1.10 (0.90–1.35)	0.349	1.20 (0.85–1.69)	0.310
Low SES	1.31 (1.12–1.53)	<0.001	1.59 (1.23–2.07)	<0.001
**Dietary score**				
cMIND	0.85 (0.82–0.88)	<0.001	0.85 (0.81–0.90)	<0.001
HEI	0.96 (0.95–0.97)	<0.001	0.96 (0.94–0.97)	<0.001
DDS	0.88 (0.85–0.90)	<0.001	0.85 (0.81–0.89)	<0.001
AIDDS	0.79 (0.75–0.83)	<0.001	0.74 (0.68–0.80)	<0.001
**Joint associations**				
**cMIND + SES**				
High diet + High SES	Ref.		Ref.	
High diet + Middle SES	1.02 (0.79–1.32)	0.876	1.05 (0.66–1.67)	0.836
High diet + Low SES	1.15 (0.95–1.40)	0.143	1.30 (0.94–1.82)	0.115
Low diet + High SES	1.56 (1.20–2.02)	<0.001	1.32 (0.85–2.05)	0.211
Low diet + Middle SES	1.70 (1.27–2.28)	<0.001	1.74 (1.09–2.78)	0.021
Low diet + Low SES	1.84 (1.52–2.21)	<0.001	2.11 (1.55–2.88)	<0.001
**HEI + SES**				
High diet + High SES	Ref.		Ref.	
High diet + Middle SES	1.02 (0.80–1.31)	0.851	1.17 (0.75–1.80)	0.49
High diet + Low SES	1.06 (0.88–1.29)	0.523	1.30 (0.94–1.80)	0.115
Low diet + High SES	1.29 (0.97–1.71)	0.078	1.46 (0.92–2.33)	0.112
Low diet + Middle SES	1.50 (1.11–2.02)	0.009	1.62 (0.98–2.65)	0.058
Low diet + Low SES	1.70 (1.42–2.03)	<0.001	2.11 (1.56–2.84)	<0.001
**DDS + SES**				
High diet + High SES	Ref.		Ref.	
High diet + Middle SES	1.06 (0.84–1.34)	0.61	1.10 (0.72–1.67)	0.664
High diet + Low SES	1.06 (0.88–1.26)	0.546	1.30 (0.96–1.76)	0.096
Low diet + High SES	1.21 (0.89–1.66)	0.227	1.36 (0.81–2.26)	0.244
Low diet + Middle SES	1.38 (1.00–1.91)	0.05	1.73 (1.04–2.88)	0.035
Low diet + Low SES	1.85 (1.54–2.22)	<0.001	2.21 (1.64–2.98)	<0.001
**AIDDS + SES**				
High diet + High SES	Ref.		Ref.	

	Depressive symptoms		Anxiety symptoms	
	**OR (95% CI)**	**P value**	**OR (95% CI)**	**P value**
High diet + Middle SES	1.01 (0.81–1.26)	0.923	1.02 (0.69–1.51)	0.902
High diet + Low SES	1.20 (1.02–1.41)	0.032	1.33 (1.01–1.76)	0.044
Low diet + High SES	1.31 (0.85–2.01)	0.222	1.03 (0.49–2.15)	0.933
Low diet + Middle SES	2.13 (1.37–3.33)	<0.001	2.34 (1.29–4.22)	0.005
Low diet + Low SES	1.92 (1.57–2.35)	<0.001	2.52 (1.86–3.42)	<0.001

Notes: This analysis used the original non-imputed dataset and included participants with complete data on all model covariates (n = 5,421). Models were adjusted for age, sex, residence, marital status, smoking, drinking, exercise, BMI, hypertension, diabetes, heart disease, stroke or other cerebrovascular disease, respiratory disease, CMMSE score, ADL disability Score, and isolation score. Continuous dietary-score estimates represent the OR per 1-point increment. For joint exposure analyses, high dietary quality + High SES was the reference group. Abbreviations: ADL, activities of daily living; AIDDS, Anti-Inflammatory Dietary Diversity Score; BMI, body mass index; CI, confidence interval; CLHLS, Chinese Longitudinal Healthy Longevity Survey; cMIND, Chinese version of the Mediterranean-DASH Intervention for Neurodegenerative Delay; CMMSE, the Chinese version of the Mini-Mental State Examination; DDS, Dietary Diversity Score; HEI, Healthy Eating Index; OR, odds ratio; SES, socioeconomic status.

Supplementary Table S6. Subgroup analyses of socioeconomic status with depressive and anxiety symptoms.

Subgroups	Depressive symptoms		Anxiety symptoms	
	OR (95% CI)	P value (FDR q)	OR (95% CI)	P value (FDR q)
<80 years				
High SES (Ref.)				
Middle SES	1.26 (0.97–1.62)	0.079 (q=0.126)	1.67 (1.13–2.47)	0.010 (q=0.019)
Low SES	1.54 (1.27–1.87)	<0.001 (q≤0.002)	1.94 (1.43–2.62)	<0.001 (q≤0.004)
≥80 years				
High SES (Ref.)				
Middle SES	1.14 (0.91–1.43)	0.249 (q=0.249)	1.20 (0.81–1.76)	0.364 (q=0.364)
Low SES	1.43 (1.21–1.70)	<0.001 (q≤0.002)	1.56 (1.17–2.07)	0.002 (q=0.005)
Male				
High SES (Ref.)				
Middle SES	1.16 (0.93–1.45)	0.179 (q=0.205)	1.22 (0.84–1.78)	0.293 (q=0.335)
Low SES	1.50 (1.26–1.78)	<0.001 (q≤0.002)	1.47 (1.09–1.97)	0.012 (q=0.019)
Female				
High SES (Ref.)				
Middle SES	1.21 (0.93–1.58)	0.154 (q=0.205)	1.59 (1.07–2.38)	0.023 (q=0.031)
Low SES	1.48 (1.23–1.78)	<0.001 (q≤0.002)	2.06 (1.54–2.77)	<0.001 (q≤0.004)

Notes: Models were adjusted for the Model 3 covariates (age, sex, residence, marital status, smoking, drinking, exercise, BMI, hypertension, diabetes, heart disease, stroke or other cerebrovascular disease, respiratory disease, CMMSE score, ADL disability Score, and isolation score), except for the subgrouping variable in the corresponding stratified analysis. Regression estimates were pooled across five multiply imputed datasets using Rubin's rules. High SES was the reference category. Abbreviations: ADL, activities of daily living; BMI, body mass index; CI, confidence interval; CMMSE, the Chinese version of the Mini-Mental State Examination; OR, odds ratio; SES, socioeconomic status. Benjamini-Hochberg false-discovery-rate (FDR) correction was applied separately within each outcome across all subgroup association tests in this table. FDR-adjusted q values are shown in parentheses after nominal P values. For nominal P values reported as <0.001, 0.001 was used conservatively in the FDR calculation; the corresponding q values are therefore conservative upper bounds.

Supplementary Table S7. Subgroup analyses of dietary quality scores with depressive and anxiety symptoms.

Subgroups	Depressive symptoms		Anxiety symptoms	
	OR (95% CI)	P value (FDR q)	OR (95% CI)	P value (FDR q)
<80 years				
cMIND	0.81 (0.78–0.85)	<0.001 (q≤0.001)	0.82 (0.77–0.87)	<0.001 (q≤0.001)
HEI	0.95 (0.94–0.96)	<0.001 (q≤0.001)	0.95 (0.93–0.96)	<0.001 (q≤0.001)
DDS	0.86 (0.83–0.89)	<0.001 (q≤0.001)	0.83 (0.79–0.88)	<0.001 (q≤0.001)
AIDDS	0.77 (0.72–0.81)	<0.001 (q≤0.001)	0.73 (0.67–0.80)	<0.001 (q≤0.001)
≥80 years				
cMIND	0.88 (0.85–0.91)	<0.001 (q≤0.001)	0.90 (0.85–0.95)	<0.001 (q≤0.001)
HEI	0.96 (0.95–0.97)	<0.001 (q≤0.001)	0.97 (0.96–0.98)	<0.001 (q≤0.001)
DDS	0.87 (0.85–0.90)	<0.001 (q≤0.001)	0.89 (0.85–0.93)	<0.001 (q≤0.001)
AIDDS	0.79 (0.75–0.83)	<0.001 (q≤0.001)	0.80 (0.74–0.87)	<0.001 (q≤0.001)
Male				
cMIND	0.85 (0.82–0.89)	<0.001 (q≤0.001)	0.88 (0.83–0.94)	<0.001 (q≤0.001)
HEI	0.95 (0.94–0.96)	<0.001 (q≤0.001)	0.97 (0.95–0.98)	<0.001 (q≤0.001)
DDS	0.85 (0.82–0.88)	<0.001 (q≤0.001)	0.90 (0.85–0.95)	<0.001 (q≤0.001)
AIDDS	0.77 (0.72–0.81)	<0.001 (q≤0.001)	0.83 (0.75–0.91)	<0.001 (q≤0.001)
Female				
cMIND	0.84 (0.81–0.87)	<0.001 (q≤0.001)	0.85 (0.80–0.89)	<0.001 (q≤0.001)
HEI	0.96 (0.95–0.97)	<0.001 (q≤0.001)	0.95 (0.94–0.97)	<0.001 (q≤0.001)
DDS	0.87 (0.85–0.90)	<0.001 (q≤0.001)	0.84 (0.80–0.87)	<0.001 (q≤0.001)
AIDDS	0.78 (0.74–0.82)	<0.001 (q≤0.001)	0.72 (0.67–0.78)	<0.001 (q≤0.001)

Notes: ORs represent the association per 1-point increment in each dietary score. Models were adjusted for the Model 3 covariates (age, sex, residence, marital status, smoking, drinking, exercise, BMI, hypertension, diabetes, heart disease, stroke or other cerebrovascular disease, respiratory disease, CMMSE score, ADL disability Score, and isolation score), except for the subgrouping variable in the corresponding stratified analysis. Regression estimates were pooled across five multiply imputed datasets using Rubin's rules. Abbreviations: ADL, activities of daily living; AIDDS, Anti-Inflammatory Dietary Diversity Score; BMI, body mass index; CI, confidence interval; cMIND, Chinese version of the Mediterranean-DASH Intervention for Neurodegenerative Delay; CMMSE, the Chinese version of the Mini-Mental State Examination; DDS, Dietary Diversity Score; HEI, Healthy Eating Index; OR, odds ratio. Benjamini-Hochberg false-discovery-rate (FDR) correction was applied separately within each outcome across all subgroup association tests in this table. FDR-adjusted q values are shown in parentheses after nominal P values. For nominal P values reported as <0.001, 0.001 was used conservatively in the FDR calculation; the corresponding q values are therefore conservative upper bounds.

Supplementary Table S8. Subgroup analyses of joint socioeconomic status-dietary quality associations with depressive and anxiety symptoms.

Subgroups	Depressive symptoms		Anxiety symptoms	
	OR (95% CI)	P value (FDR q)	OR (95% CI)	P value (FDR q)
<80 years				
cMIND + SES				
High diet + Middle SES	1.04 (0.76–1.42)	0.806 (q=0.860)	1.49 (0.92–2.42)	0.105 (q=0.163)
High diet + Low SES	1.19 (0.95–1.50)	0.132 (q=0.220)	1.45 (1.00–2.10)	0.049 (q=0.093)
Low diet + High SES	1.59 (1.10–2.29)	0.014 (q=0.040)	1.32 (0.73–2.37)	0.358 (q=0.434)
Low diet + Middle SES	2.33 (1.59–3.41)	<0.001 (q≤0.004)	2.37 (1.39–4.06)	0.002 (q=0.008)
Low diet + Low SES	2.30 (1.84–2.87)	<0.001 (q≤0.004)	2.61 (1.85–3.69)	<0.001 (q≤0.004)
HEI + SES				
High diet + Middle SES	0.99 (0.73–1.35)	0.956 (q=0.958)	1.50 (0.92–2.45)	0.100 (q=0.163)
High diet + Low SES	1.22 (0.97–1.53)	0.090 (q=0.153)	1.58 (1.09–2.30)	0.017 (q=0.0504)
Low diet + High SES	1.22 (0.82–1.81)	0.33 (q=0.412)	1.90 (1.06–3.39)	0.031 (q=0.069)
Low diet + Middle SES	2.33 (1.56–3.46)	<0.001 (q≤0.004)	2.91 (1.69–5.00)	<0.001 (q≤0.004)
Low diet + Low SES	1.97 (1.58–2.45)	<0.001 (q≤0.004)	2.74 (1.94–3.87)	<0.001 (q≤0.004)
DDS + SES				
High diet + Middle SES	1.11 (0.83–1.48)	0.491 (q=0.578)	1.69 (1.08–2.64)	0.022 (q=0.0550)
High diet + Low SES	1.21 (0.97–1.50)	0.087 (q=0.151)	1.52 (1.07–2.16)	0.018 (q=0.0514)
Low diet + High SES	0.94 (0.61–1.45)	0.779 (q=0.842)	1.44 (0.75–2.75)	0.272 (q=0.340)
Low diet + Middle SES	1.75 (1.15–2.67)	0.009 (q=0.027)	2.06 (1.12–3.79)	0.020 (q=0.0516)
Low diet + Low SES	2.03 (1.63–2.52)	<0.001 (q≤0.004)	2.71 (1.93–3.81)	<0.001 (q≤0.004)
AIDDS + SES				
High diet + Middle SES	1.05 (0.77–1.45)	0.744 (q=0.815)	1.67 (1.01–2.76)	0.046 (q=0.092)
High diet + Low SES	1.15 (0.91–1.46)	0.235 (q=0.336)	1.42 (0.96–2.11)	0.079 (q=0.134)
Low diet + High SES	1.13 (0.81–1.59)	0.470 (q=0.561)	1.67 (0.99–2.83)	0.054 (q=0.098)
Low diet + Middle SES	1.83 (1.26–2.66)	0.002 (q=0.007)	2.40 (1.41–4.11)	0.001 (q=0.004)
Low diet + Low SES	2.02 (1.62–2.53)	<0.001 (q≤0.004)	2.90 (2.03–4.15)	<0.001 (q≤0.004)
≥80 years				
cMIND + SES				
High diet + Middle SES	0.99 (0.77–1.28)	0.958 (q=0.958)	1.04 (0.65–1.66)	0.880 (q=0.891)
High diet + Low SES	1.21 (1.00–1.47)	0.050 (q=0.111)	1.44 (1.03–2.00)	0.031 (q=0.069)
Low diet + High SES	0.91 (0.65–1.27)	0.585 (q=0.659)	1.23 (0.71–2.13)	0.468 (q=0.510)

Subgroups	Depressive symptoms		Anxiety symptoms	
	OR (95% CI)	P value (FDR q)	OR (95% CI)	P value (FDR q)
Low diet + Middle SES	1.60 (1.10–2.34)	0.015 (q=0.041)	1.88 (1.06–3.34)	0.030 (q=0.069)
Low diet + Low SES	1.76 (1.44–2.14)	<0.001 (q≤0.004)	1.91 (1.37–2.66)	<0.001 (q≤0.004)
HEI + SES				
High diet + Middle SES	1.12 (0.86–1.45)	0.404 (q=0.497)	1.12 (0.71–1.79)	0.625 (q=0.649)
High diet + Low SES	1.20 (0.98–1.46)	0.074 (q=0.141)	1.43 (1.02–2.00)	0.039 (q=0.084)
Low diet + High SES	1.20 (0.86–1.68)	0.289 (q=0.379)	1.28 (0.73–2.25)	0.386 (q=0.441)
Low diet + Middle SES	1.38 (0.96–1.98)	0.084 (q=0.151)	1.61 (0.90–2.88)	0.108 (q=0.163)
Low diet + Low SES	1.82 (1.50–2.22)	<0.001 (q≤0.004)	1.86 (1.34–2.58)	<0.001 (q≤0.004)
DDS + SES				
High diet + Middle SES	1.17 (0.90–1.51)	0.250 (q=0.345)	1.20 (0.74–1.93)	0.455 (q=0.507)
High diet + Low SES	1.26 (1.03–1.53)	0.022 (q=0.0550)	1.50 (1.07–2.11)	0.019 (q=0.0516)
Low diet + High SES	1.46 (1.06–2.03)	0.022 (q=0.0550)	1.78 (1.06–2.99)	0.030 (q=0.069)
Low diet + Middle SES	1.42 (0.99–2.03)	0.057 (q=0.120)	1.77 (1.00–3.14)	0.050 (q=0.093)
Low diet + Low SES	1.96 (1.60–2.39)	<0.001 (q≤0.004)	2.11 (1.51–2.95)	<0.001 (q≤0.004)
AIDDS + SES				
High diet + Middle SES	1.08 (0.85–1.38)	0.526 (q=0.610)	1.16 (0.75–1.78)	0.511 (q=0.538)
High diet + Low SES	1.30 (1.08–1.56)	0.005 (q=0.017)	1.39 (1.01–1.89)	0.040 (q=0.084)
Low diet + High SES	1.25 (0.82–1.89)	0.298 (q=0.385)	1.35 (0.70–2.62)	0.370 (q=0.435)
Low diet + Middle SES	1.71 (1.08–2.72)	0.022 (q=0.0550)	1.66 (0.83–3.30)	0.152 (q=0.210)
Low diet + Low SES	2.03 (1.65–2.49)	<0.001 (q≤0.004)	2.19 (1.58–3.03)	<0.001 (q≤0.004)
Male				
cMIND + SES				
High diet + Middle SES	0.92 (0.69–1.22)	0.543 (q=0.621)	0.98 (0.58–1.64)	0.937 (q=0.937)
High diet + Low SES	1.14 (0.91–1.42)	0.246 (q=0.345)	1.31 (0.90–1.92)	0.163 (q=0.221)
Low diet + High SES	1.17 (0.88–1.56)	0.279 (q=0.372)	1.11 (0.67–1.83)	0.679 (q=0.696)
Low diet + Middle SES	1.76 (1.30–2.40)	<0.001 (q≤0.004)	1.64 (1.01–2.67)	0.048 (q=0.093)
Low diet + Low SES	1.93 (1.57–2.36)	<0.001 (q≤0.004)	1.65 (1.17–2.34)	0.005 (q=0.017)
HEI + SES				
High diet + Middle SES	0.99 (0.76–1.30)	0.942 (q=0.958)	1.20 (0.75–1.91)	0.456 (q=0.507)
High diet + Low SES	1.21 (0.97–1.51)	0.086 (q=0.151)	1.34 (0.91–1.96)	0.135 (q=0.196)
Low diet + High SES	1.41 (1.04–1.93)	0.029 (q=0.070)	1.39 (0.82–2.34)	0.218 (q=0.281)

Subgroups	Depressive symptoms		Anxiety symptoms	
	OR (95% CI)	P value (FDR q)	OR (95% CI)	P value (FDR q)
Low diet + Middle SES	1.89 (1.36–2.61)	<0.001 (q≤0.004)	1.56 (0.92–2.65)	0.102 (q=0.163)
Low diet + Low SES	1.94 (1.59–2.37)	<0.001 (q≤0.004)	1.75 (1.24–2.46)	0.001 (q=0.004)
DDS + SES				
High diet + Middle SES	1.02 (0.79–1.32)	0.878 (q=0.924)	1.34 (0.86–2.10)	0.196 (q=0.257)
High diet + Low SES	1.25 (1.02–1.53)	0.031 (q=0.073)	1.42 (0.99–2.03)	0.055 (q=0.098)
Low diet + High SES	1.29 (0.92–1.81)	0.145 (q=0.227)	1.87 (1.11–3.17)	0.020 (q=0.0516)
Low diet + Middle SES	1.85 (1.30–2.63)	<0.001 (q≤0.004)	1.53 (0.86–2.74)	0.152 (q=0.210)
Low diet + Low SES	2.02 (1.65–2.48)	<0.001 (q≤0.004)	1.96 (1.39–2.78)	<0.001 (q≤0.004)
AIDDS + SES				
High diet + Middle SES	0.85 (0.64–1.13)	0.271 (q=0.367)	1.25 (0.77–2.03)	0.370 (q=0.435)
High diet + Low SES	1.18 (0.95–1.48)	0.138 (q=0.225)	1.25 (0.84–1.85)	0.268 (q=0.340)
Low diet + High SES	1.28 (0.97–1.68)	0.082 (q=0.151)	1.29 (0.80–2.07)	0.293 (q=0.361)
Low diet + Middle SES	2.21 (1.60–3.06)	<0.001 (q≤0.004)	1.45 (0.85–2.45)	0.170 (q=0.227)
Low diet + Low SES	1.99 (1.62–2.45)	<0.001 (q≤0.004)	1.82 (1.27–2.59)	<0.001 (q≤0.004)
Female				
cMIND + SES				
High diet + Middle SES	1.21 (0.90–1.63)	0.201 (q=0.298)	1.50 (0.94–2.39)	0.090 (q=0.150)
High diet + Low SES	1.34 (1.09–1.64)	0.006 (q=0.019)	1.79 (1.28–2.49)	<0.001 (q≤0.004)
Low diet + High SES	1.49 (1.00–2.24)	0.052 (q=0.112)	1.26 (0.67–2.37)	0.472 (q=0.510)
Low diet + Middle SES	1.65 (1.03–2.65)	0.037 (q=0.085)	2.27 (1.20–4.29)	0.012 (q=0.037)
Low diet + Low SES	2.11 (1.70–2.62)	<0.001 (q≤0.004)	2.82 (2.01–3.96)	<0.001 (q≤0.004)
HEI + SES				
High diet + Middle SES	1.21 (0.89–1.63)	0.223 (q=0.324)	1.48 (0.92–2.38)	0.107 (q=0.163)
High diet + Low SES	1.16 (0.94–1.43)	0.161 (q=0.248)	1.77 (1.26–2.49)	0.001 (q=0.004)
Low diet + High SES	1.19 (0.79–1.81)	0.410 (q=0.497)	1.61 (0.85–3.04)	0.144 (q=0.206)
Low diet + Middle SES	1.40 (0.89–2.19)	0.142 (q=0.227)	2.55 (1.39–4.68)	0.003 (q=0.011)
Low diet + Low SES	2.05 (1.66–2.53)	<0.001 (q≤0.004)	2.84 (2.03–3.97)	<0.001 (q≤0.004)
DDS + SES				
High diet + Middle SES	1.33 (0.98–1.80)	0.066 (q=0.132)	1.47 (0.91–2.37)	0.114 (q=0.169)
High diet + Low SES	1.21 (0.98–1.49)	0.072 (q=0.140)	1.65 (1.18–2.32)	0.004 (q=0.014)
Low diet + High SES	1.23 (0.83–1.83)	0.305 (q=0.387)	1.33 (0.71–2.50)	0.375 (q=0.435)

Subgroups	Depressive symptoms		Anxiety symptoms	
	**OR (95% CI)**	**P value (FDR q)**	**OR (95% CI)**	**P value (FDR q)**
Low diet + Middle SES	1.10 (0.71–1.71)	0.659 (q=0.732)	2.29 (1.25–4.18)	0.007 (q=0.022)
Low diet + Low SES	1.99 (1.61–2.46)	<0.001 (q≤0.004)	2.80 (2.01–3.91)	<0.001 (q≤0.004)
AIDDS + SES				
High diet + Middle SES	1.31 (0.98–1.73)	0.064 (q=0.131)	1.56 (1.01–2.41)	0.044 (q=0.090)
High diet + Low SES	1.31 (1.08–1.59)	0.007 (q=0.022)	1.71 (1.25–2.34)	<0.001 (q≤0.004)
Low diet + High SES	1.44 (0.83–2.52)	0.197 (q=0.297)	1.33 (0.57–3.11)	0.504 (q=0.538)
Low diet + Middle SES	0.98 (0.56–1.74)	0.957 (q=0.958)	2.07 (0.95–4.48)	0.066 (q=0.115)
Low diet + Low SES	2.28 (1.83–2.84)	<0.001 (q≤0.004)	3.25 (2.34–4.51)	<0.001 (q≤0.004)

Notes: Dietary scores were dichotomized as high or low using the median within each imputed dataset. High dietary quality + High SES was the reference category for each dietary index and is not shown. Models were adjusted for the Model 3 covariates (age, sex, residence, marital status, smoking, drinking, exercise, BMI, hypertension, diabetes, heart disease, stroke or other cerebrovascular disease, respiratory disease, CMMSE score, ADL disability Score, and isolation score), except for the subgrouping variable in the corresponding stratified analysis. Regression estimates were pooled across five multiply imputed datasets using Rubin's rules. Abbreviations: ADL, activities of daily living; AIDDS, Anti-Inflammatory Dietary Diversity Score; BMI, body mass index; CI, confidence interval; cMIND, Chinese version of the Mediterranean-DASH Intervention for Neurodegenerative Delay; CMMSE, the Chinese version of the Mini-Mental State Examination; DDS, Dietary Diversity Score; HEI, Healthy Eating Index; OR, odds ratio; SES, socioeconomic status. Benjamini-Hochberg false-discovery-rate (FDR) correction was applied separately within each outcome across all subgroup association tests in this table. FDR-adjusted q values are shown in parentheses after nominal P values. For nominal P values reported as <0.001, 0.001 was used conservatively in the FDR calculation; the corresponding q values are therefore conservative upper bounds.

Supplementary Table S9. Multiplicative interactions between socioeconomic status and dichotomized dietary quality in relation to depressive and anxiety symptoms.

Dietary index	Middle SES × low dietary quality, OR (95% CI)	P value	Low SES × low dietary quality, OR (95% CI)	P value	Overall P for interaction (FDR q)
**Panel A. Depressive symptoms**					
cMIND	1.28 (0.88-1.86)	0.199	1.16 (0.90-1.50)	0.265	0.395 (q=0.632)
HEI	1.30 (0.87-1.95)	0.207	1.23 (0.92-1.65)	0.167	0.340 (q=0.632)
DDS	1.07 (0.72-1.58)	0.750	1.28 (0.97-1.68)	0.084	0.147 (q=0.588)
AIDDS	1.42 (0.85-2.36)	0.180	1.26 (0.87-1.81)	0.219	0.363 (q=0.632)
**Panel B. Anxiety symptoms**					
cMIND	1.95 (1.08-3.53)	0.027	1.51 (0.98-2.34)	0.063	0.074 (q=0.588)
HEI	0.99 (0.53-1.83)	0.971	0.95 (0.60-1.50)	0.820	0.964 (q=0.964)
DDS	0.80 (0.44-1.46)	0.471	0.97 (0.63-1.48)	0.873	0.719 (q=0.822)
AIDDS	1.25 (0.59-2.64)	0.559	1.30 (0.74-2.29)	0.355	0.649 (q=0.822)

Notes: High SES and high dietary quality were the reference categories. Dietary scores were dichotomized at the median within each imputed dataset. Each model included SES, dichotomized dietary quality, and the SES × dietary quality interaction terms and was adjusted for age, sex, residence, marital status, smoking, drinking, exercise, BMI, hypertension, diabetes, heart disease, stroke or other cerebrovascular disease, respiratory disease, CMMSE score, ADL disability Score, and isolation score. The reported interaction ORs are exponentiated coefficients for the corresponding multiplicative interaction terms. Overall P for interaction represents a joint test of the two SES × dietary quality interaction terms. Regression estimates were pooled across five multiply imputed datasets using Rubin's rules. Abbreviations: ADL, activities of daily living; AIDDS, Anti-Inflammatory Dietary Diversity Score; BMI, body mass index; CI, confidence interval; cMIND, Chinese version of the Mediterranean-DASH Intervention for Neurodegenerative Delay; CMMSE, the Chinese version of the Mini-Mental State Examination; DDS, Dietary Diversity Score; HEI, Healthy Eating Index; OR, odds ratio; SES, socioeconomic status. Benjamini-Hochberg false-discovery-rate (FDR) correction was applied across the eight overall interaction tests in this table (four dietary indices × two outcomes). The individual product-term P values are shown for model interpretation but the multiplicity-adjusted inference is based on the overall interaction tests. FDR-adjusted q values are shown in parentheses after the overall P values.

Supplementary Table S10. Multiplicative interactions between socioeconomic status and standardized continuous dietary quality scores in relation to depressive and anxiety symptoms.

Dietary index	Middle SES × dietary score (per 1-SD), OR (95% CI)	P value	Low SES × dietary score (per 1-SD), OR (95% CI)	P value	Overall P for interaction (FDR q)
**Panel A. Depressive symptoms**					
cMIND	0.90 (0.75-1.07)	0.238	0.94 (0.84-1.07)	0.353	0.465 (q=0.685)
HEI	0.84 (0.70-1.00)	0.056	0.86 (0.75-0.97)	0.015	0.041 (q=0.109)
DDS	0.86 (0.71-1.03)	0.091	0.78 (0.69-0.88)	<0.001	<0.001 (q≤0.008)
AIDDS	0.84 (0.70-0.99)	0.039	0.83 (0.73-0.93)	0.001	0.005 (q=0.020)
**Panel B. Anxiety symptoms**					
cMIND	0.87 (0.66-1.17)	0.360	0.92 (0.75-1.12)	0.380	0.599 (q=0.685)
HEI	0.93 (0.70-1.24)	0.604	0.95 (0.77-1.16)	0.598	0.843 (q=0.843)
DDS	1.00 (0.75-1.33)	0.975	0.91 (0.74-1.12)	0.359	0.531 (q=0.685)
AIDDS	1.00 (0.76-1.31)	0.999	0.91 (0.75-1.10)	0.331	0.497 (q=0.685)

Notes: High SES was the reference category. Each dietary score was standardized to a mean of 0 and an SD of 1 before model fitting. Each model included SES, the standardized dietary score, and the SES × dietary score interaction terms and was adjusted for age, sex, residence, marital status, smoking, drinking, exercise, BMI, hypertension, diabetes, heart disease, stroke or other cerebrovascular disease, respiratory disease, CMMSE score, ADL disability Score, and isolation score. The reported interaction ORs are exponentiated coefficients for the corresponding multiplicative interaction terms per 1-SD increment in the dietary score. Overall P for interaction represents a joint test of the two SES × dietary score interaction terms. Regression estimates were pooled across five multiply imputed datasets using Rubin's rules. Abbreviations: ADL, activities of daily living; AIDDS, Anti-Inflammatory Dietary Diversity Score; BMI, body mass index; CI, confidence interval; cMIND, Chinese version of the Mediterranean-DASH Intervention for Neurodegenerative Delay; CMMSE, the Chinese version of the Mini-Mental State Examination; DDS, Dietary Diversity Score; HEI, Healthy Eating Index; OR, odds ratio; SD, standard deviation; SES, socioeconomic status. Benjamini-Hochberg false-discovery-rate (FDR) correction was applied across the eight overall interaction tests in this table (four dietary indices × two outcomes). The individual product-term P values are shown for model interpretation but the multiplicity-adjusted inference is based on the overall interaction tests. For the overall DDS-depression P value reported as <0.001, 0.001 was used conservatively in the FDR calculation, so its q value is a conservative upper bound.
